# miR156f integrates panicle architecture through genetic modulation of branch number and pedicel length pathways

**DOI:** 10.1186/s12284-019-0299-5

**Published:** 2019-05-30

**Authors:** Xiaofang Yang, Jiang Wang, Zhengyan Dai, Xiaoling Zhao, Xuexia Miao, Zhenying Shi

**Affiliations:** 1 Key Laboratory of Insect Developmental and Evolutionary Biology, Institute of Plant Physiology and Ecology, Shanghai Institutes for Biological Sciences, Chinese Academy of Sciences, Shanghai, 200032 China; 2 University of Chinese Academy of Sciences, Shanghai, 200032 China; 3 National Key Laboratory of Plant Molecular Genetics, Institute of Plant Physiology and Ecology, Shanghai Institutes for Biological Sciences, the Chinese Academy of Sciences, Shanghai, 200032 China

**Keywords:** *Oryza sativa*, miR156, Panicle development, *LAX1*, *LAX2*, *RCN2*, *OsRA2*

## Abstract

**Background:**

Rice (*Oryza sativa*) panicle architecture is the major determinant of the ideal plant architecture that directly influence yield potential. Many genes influencing development of primary branches, secondary branches, spikelet and pedicel would also influence panicle architecture, which is thus a complex trait regulated by genes from various aspects. miR156, an extensively studied miRNA, has recently emerged as promising target for crop improvement because of its role in plant architecture regulation, such as the number of tillers, plant height and the panicle architecture. Increasing evidence suggests that miR156 might play an important role in panicle architecture regulation.

**Main body:**

To study the detailed function of miR156 in rice panicle architecture regulation, we examined the genetic interaction or transcriptional regulation of miR156/OsSPL to other panicle regulating genes. Our results revealed that expression of many panicle related genes were influenced by miR156. Through biochemical analysis, we further proved that miR156 directly regulated the axillary meristem regulating gene, *LAX1*, at the transcription level. And the intimate relations between miR156 and *LAX1*, and miR156 and *LAX2* were also uncovered by genetic analysis. On the other hand, a tight genetic linkage between miR156 and *RCN2*, the panicle branch promoting gene, was also detected, which suggested a buffering mechanism for the miR156 mediated panicle architecture regulation. Furthermore, genetic analysis also demonstrated that miR156 functioned in the same pathway with *OsRA2* to regulate pedicel length.

**Short conclusion:**

Altogether, miR156 integrates several genetic pathways mediated by genes such as *LAX1*, *LAX2*, *RCN2* and *OsRA2*, and comprehensively regulates panicle development in rice. Based on these analysis, we concluded that miR156 acts as an important regulator for panicle architecture through influencing various aspects of panicle development.

**Electronic supplementary material:**

The online version of this article (10.1186/s12284-019-0299-5) contains supplementary material, which is available to authorized users.

## Background

As one of the most important cereal crops, rice (*Oryza sativa*) provides food for more than half of the world population. And still greater challenge would be faced to meet the need of the increasing population under condition such as shrunk arable land, changing climate and less water (Wang et al. 2018). Increasing world population calls for high yield from crop plants, therefore, factors that affect rice yield always attract the major research attention. Panicle architecture (inflorescence patterning) is one of the specific morphological characters of rice, which comprehensively coordinates panicle length, the primary branches (PB) and secondary branches (SB), the spikelet, and pedicel length (Itoh et al. 2005). In agriculture, panicle architecture is the major determinant of the ideal plant architecture that directly influence rice yield potential. Therefore, many studies have focused on the genes associated with the panicle development and the underlying mechanism.

Panicle architecture is mainly determined by the timing of identity transition of different type of meristems (Kyozuka et al. 2014). Accordingly, genes functioning in the temporal control of the meristem phase affect panicle development. Quite a few factors involved in phase transition and inflorescence have been studied in *Arabidopsis*, and function of several homologous genes has been revealed through comparative study in rice. Two of the four rice *RCN* genes regulated the transition from shoot apical meristem (SAM) to inflorescence meristem (IM), when overexpressed, the plants showed greatly increased PBs and SBs in the panicle (Nakagawa et al. 2002). The homologous gene of *RCN* in *Arabidopsis* and *Antirrhinum* respectively, also function in meristem transition (Bradley et al. 1997; Bradley et al. 1996). Rice *TERMINAL FLOWER 1 (TFL1)/CENTRORADIALIS (CEN)* like genes enhance the number of branches in the panicle by promoting the activity of secondary meristems (Zhang et al. 2005). *TAWAWA1* positively regulates the branch number in the panicle through delaying the transition of IM to spikelet meristem (SM) (Yoshida et al. 2013). And *OsRAMOSA2* (*OsRA2*) expressed in the meristem of the PBs and SBs in the panicle regulates pedicel length, a variable function from its homologues gene *RAMOSA2* in maize, which showed increased branches when mutated (Lu et al. 2017; Bortiri et al. 2006).

Meanwhile, the architecture of the whole plants, whether at vegetative stage or reproductive stage, is determined by the activity of the shoot apical meristem (SAM) and axillary meristem (AM)*. LAX PANICLE 1* (*LAX1*), *LAX2* and *MONOCULM1*(*MOC1*) control rice axillary development and influenced both the number of tillers in vegetative stage and the number of branches in the panicle (Komatsu et al. 2003; Tabuchi et al. 2011; Li et al. 2003). Furthermore, *LAX1* and *LAX2* function synergistically in regulating axillary development (Tabuchi et al. 2011). *GHD8* could influence the number of branches by regulating *MOC1* (Yan et al. 2011). *FRIZZY PANICLE* (*FZP*) restrains overgrowth of AM and results in excessive ramification of rachis-branches when mutated (Komatsu et al. 2001). *LARGER PANICLE* (*LP*) gene expresses in the branch meristems and positively regulates the number of branches in the panicle (Li et al. 2011). Meanwhile, some plant hormones also function in branching, or axillary development (McSteen 2009). Genes involved in plant hormone biosynthesis or signaling also influence panicle development. For example, phytohormone cytokinin (CK) has positive effect on meristem activity and maintenance, with the up-regulation of the *CYTOKININ OXIDASE* (*OsCKX2*) in CK signaling pathway resulting in increased PBs and SBs (Ashikari et al. 2005). DROUGHT AND SALT TOLERANCE (DST) protein also influenced the number of branches in the panicle through regulation on *OsCKX2* (Li et al. 2013). However, gibberellin (GA) signaling might negatively regulate the number of branches in the panicle through antagonistic crosstalk with CK (Wu et al. 2016).

microRNAs (miRNAs) have emerged as a new force in regulating plant development and physiology, increasing evidence suggests them to be coordinated integrator of complex traits, with the potential in crop improvement (Tang and Chu 2017; Wang et al. 2015). Ever since the clarification of its role in regulating developmental timing, miR156 and its targets are extensively studied (Wu et al. 2009). In rice, the miR156/*SQUAMOSA PROMOTER BINDING PROTEIN-like* (*SPL*) module proves to be good target for crop improvement for its role in plant architecture regulation, such as the number of tillers, plant height and the panicle architecture (Wang and Wang 2015; Wang and Zhang 2017). Specifically, *OsSPL14* positively regulates the number of PBs in the panicle (Jiao et al. 2010; Miura et al. 2010). And generally, miR156 regulates plant height and tiller number in rice (Dai et al. 2018; Xie et al. 2006). In regulating panicle development, miR156 is revealed to regulate the coordinated development of the branching in vegetative and reproductive stage, together with several other miRNAs and factors (Wang et al. 2015). However, since panicle development is a complex processes involving multiple pathways and many regulating factors with the underlying crosstalk less revealed, still many genetic relations needs to be studied to further understand the genetic regulation of panicle development. Although miR156 is a vegetative specific factor, the target genes of miR156, *OsSPLs*, function in the reproductive stage, indicating the possible function of miR156 in panicle traits determination that remains to be further revealed.

In this study, we proved that miR156 regulated panicle development, with the *cs* mutant plants in which miR156f was over expressed showing small panicle, and MIM156fOE plants in which miR156 was down-regulated showing increased panicle length and decreased number of SBs, and expression of many genes functioned in panicle regulation was influenced by miR156. In genetic analysis, we revealed the possible relation between miR156 and *LAX1*, *LAX2*, *RCN2* and *OsRA2*. Through these analysis, we investigated the possible pathways through which miR156 regulated axillary development, the number of branches, and pedicel development in the panicle.

## Main text

### Sequestering miR156 resulted in sparse panicle with less SBs and longer pedicel

**Fig. 1:**
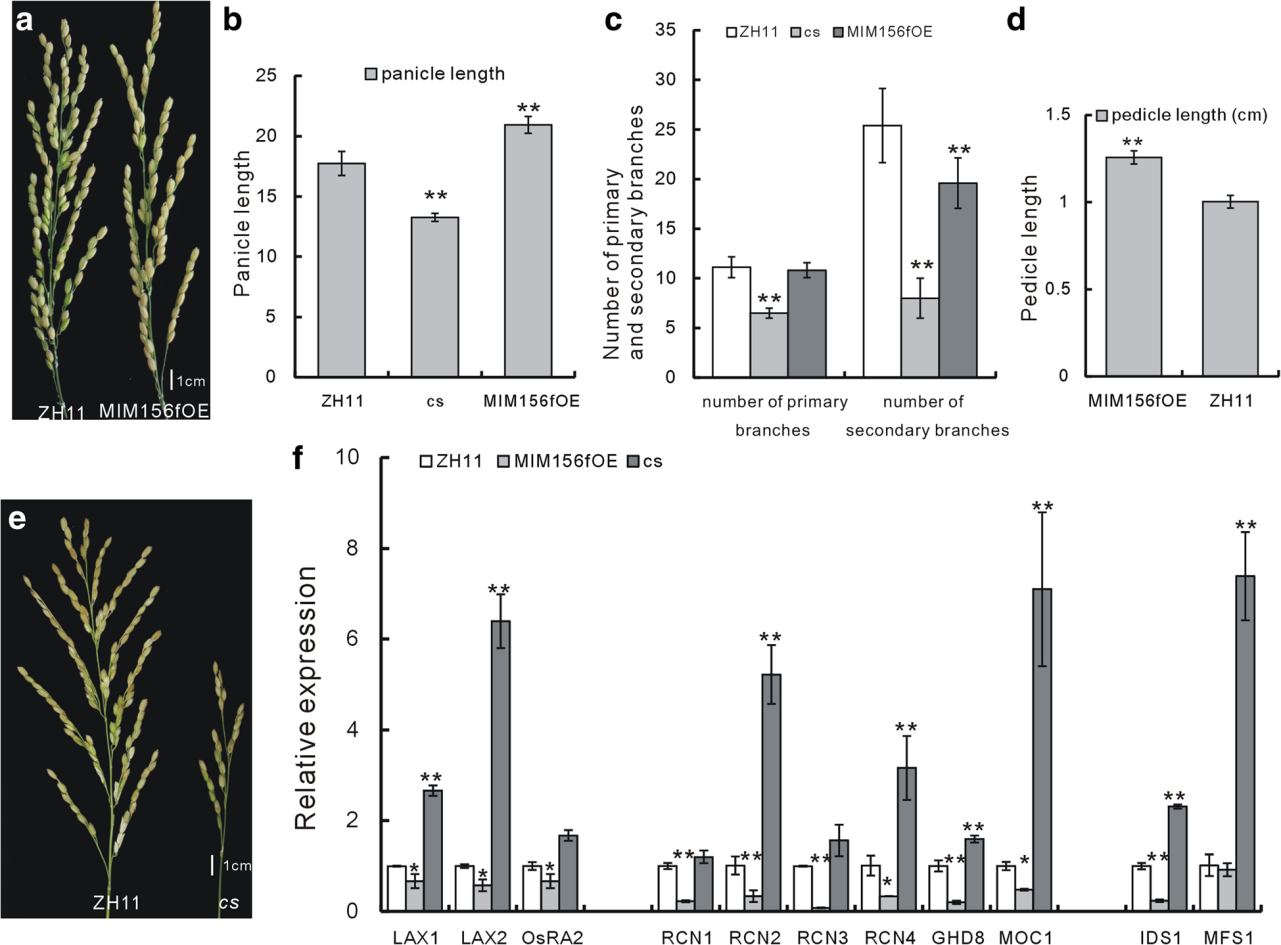
Panicle characters of the miR156f-related plants and expression analysis of some panicle related genes. **a** Overall morphology of the panicles of the MIM156fOE and the WT ZH11 plants. **b** Statistical analysis of the panicle length of the MIM156fOE plants, the *cs* mutant and the WT ZH11 (*n* = 30). **c** Statistical analysis of the number of the PBs and SBs of the MIM156fOE plants, the *cs* mutant and the WT ZH11 (*n* = 20). **d** Statistical analysis of the pedicel length of the MIM156fOE plants and the WT ZH11 (*n* = 20). **e** Overall morphology of the panicles of the *cs* mutant and the WT ZH11. **f** Expression analysis of some panicle development related genes in the MIM156fOE plants, the *cs* mutant and the WT ZH11 by qRT-PCR (*n* = 3), young panicles of 2 cm long were used for detection. Single and double asterisks represent significant difference determined by the Student’s *t*-test at *P < 0.05 and **P < 0.01

In our previous study, it was proved that miR156f regulated plant height and tiller number in rice through the auxin signaling pathway (Dai et al. 2018). Furthermore, we found that miR156f also had effects on the panicle morphology. Specifically, the constitutively expressed MIM156f in the MIM156fOE transgenic plants, sequestered the native miR156, and induced sparse panicles (Fig. 1a). The panicle architecture related characteristics in the MIM156fOE plants such as the panicle was longer, the number of the SBs but not the PBs was decreased, as compared with those in the wild type (WT) plants (Fig. 1a, b, c). These changes on the panicle indicated that the transition from SBs to spikelets was affected when miR156 was down-regulated. In addition, the pedicels of the MIM156fOE plants were also elongated as compared with that of the WT (Fig. 1d). Altogether, these morphological features of the panicle changes made the panicle in the MIM156fOE plants a sparse phenotype.

Further, we had isolated a T-DNA insertion mutant named *cs* mutant, in which the expression of miR156f was significantly up-regulated (Dai et al. 2018). The *cs* mutant had a smaller and shortened panicle as compared with the WT (Fig. 1b, e), accompanied with less PBs and SBs (Fig. 1c), these characters are in consistence with the significantly decreased plant height of the *cs* mutant (Dai et al. 2018). In contrast to the panicle changes in the mutant, the pedicel length showed no significant difference as compared with that of the WT plants (data not shown).

In summary, changes of the morphological features of in the panicle of the MIM156fOE plants and the *cs* mutant indicated that although miR156 is extensively studied as a vegetative factor, it was also involved in panicle architecture regulation in rice.

### miR156f regulated the expression level of genes related to panicle development

We have shown that miR156f played a role in panicle development. Further, we wanted to know if its function in the panicle was mediated by regulation on the panicle related genes. The microarray data of the *cs* mutant (Dai et al. 2018) revealed that the expression level of several panicle development related genes, such as *LAX2*, *RCN1*, *RCN3* and *OsCKX2* was greatly changed compared with WT (Table 1).

**Table 1:** Expression of some panicle related genes in the microarray data of the *cs* mutant

Gene names and ID	Log2 fold change (“+”indicats up-egualtion; “_” indicates down-regulation)
LOC_Os06g40780(*MOC1*)	−1.5
LOC_Os01g61480(*LAX1*)	+ 1.0
LOC_Os04g32510(*LAX2*)	−2.0
LOC_ Os11g05470(*RCN1*)	+ 7.5
LOC_Os12g05590(*RCN3*)	+ 4.7
LOC_ Os04g33570(*RCN4*)	−1.2
LOC_Os01g07480(*OsRA2*)	+ 1.3
LOC_Os08g39890(*OsSPL14*)	+ 1.3
LOC_Os03g60430(*OsIDS1*)	−1.7
LOC_Os05g41760(*MFS1*)	−1.4
LOC_Os08g07740(*GHD8*)	+ 1.1
LOC_Os01g10110(*OsCKX2*)	+ 2.6

The expression level of selected panicle development related genes in the MIM156fOE plants and the *cs* mutant was analyzed by quantitative real-time PCR (qRT-PCR). Genes detected included those involved in axillary meristems development, such as *LAX1*(Oikawa and Kyozuka 2009; Komatsu et al. 2003) and *LAX2* (Tabuchi et al. 2011), pedicel length regulation, such as *OsRA2* (Lu et al. 2017), those regulating PB and SB development, such as *RCN1*, *RCN2*, *RCN3*, *RCN4* (Nakagawa et al. 2002), *GHD8* (Yan et al. 2011) and *MOC1*(Li et al. 2003), and those in spikelet development, such as *MFS1* and *OsIDS1*(Ren et al. 2013; Lee and An 2012). It was revealed that the expression level of these selected genes was significantly changed by miR156f over expression (the *cs* mutant) and/or miR156 down regulation (the MIM156fOE plants) when compared with WT plants (Fig. 1f). This analysis further indicated the possible involvement of miR156f in panicle development.

### Genetic analysis revealed interaction between miR156f and *LAX1*

**Fig. 2:**
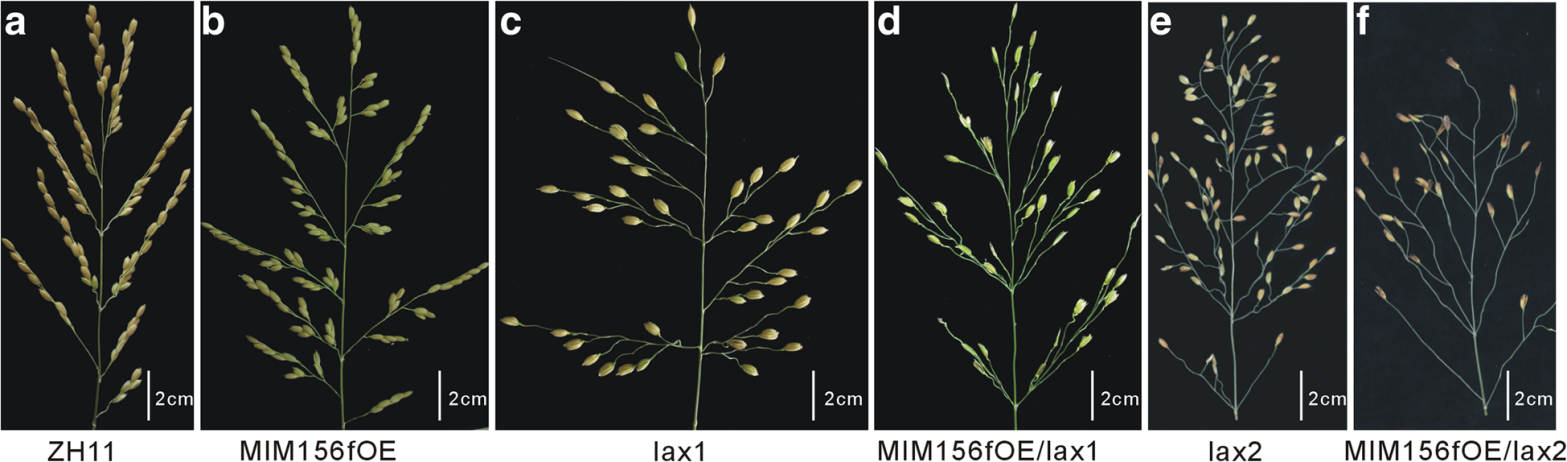
Panicle phenotype of the MIM156fOE/lax1 hybrid and the MIM156fOE/lax2 hybrid. **a** ZH11, **b** MIM156fOE, **c** *lax1*, **d** MIM156fOE*/lax1*, **e** *lax2*, **f** MIM156fOE*/lax2*

In rice, *LAX1* gene is responsible for AM initiation and maintenance. The *lax1* mutant showed reduced high-order branches and spikelets, which was due to the defects in initiation or maintenance of the lateral panicle development (Komatsu et al. 2003). To investigate the potential interaction between miR156 and *LAX1*, we crossed the MIM156fOE plant with the *lax1* mutant, a natural genetic mutant in the ZH11 background. The positive F2 hybrid showed sparser panicle, much less number of SBs and sterile spikelets (Fig. 2d) compared with the parents (Fig. 2b, c) and the WT (Fig. 2a), but no change of the number of PBs. This synergistically enhancement on the panicle defects indicated the possible genetic interaction between miR156 and *LAX1*.

### SPL protein could directly regulate *LAX1* expression at the transcription level

The genetic analysis had confirmed the interaction between miR156f and *LAX1* in panicle development, and then we wanted to test whether *LAX1* could be regulated by miR156 at the transcriptional level. Firstly, the expression level of *LAX1* in the MIM156fOE plants and the *cs* mutant was analyzed. It showed that the expression of *LAX1* was down-regulated in the MIM156fOE plants, while up-regulated in the *cs* mutant (Fig. 1f). This confirmed that *LAX1* gene was affected by miR156 at the transcriptional level.

**Fig. 3:**
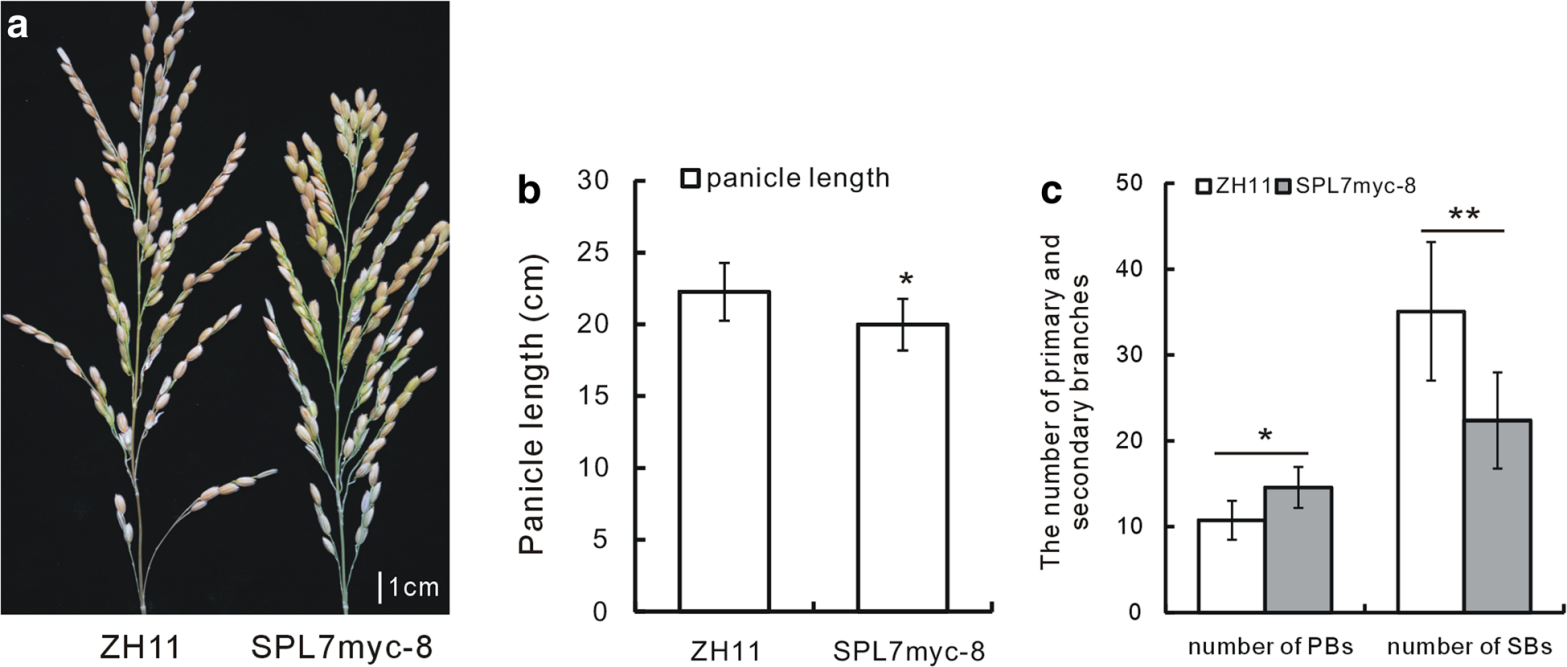
Panicle characters of the SPL7myc-8 line. **a** Morphology of the panicles of the WT and the SPL7myc-8 line. **b** Statistical analysis of the panicle length of the WT and the SPL7myc-8 line (*n* = 20). **c** Statistical analysis of the number of the PBs and SBs of the WT and the SPL7myc-8 line (*n* = 20). Single and double asterisks represent significant difference determined by the Student’s *t*-test at *P < 0.05 and **P < 0.01

**Fig. 4:**
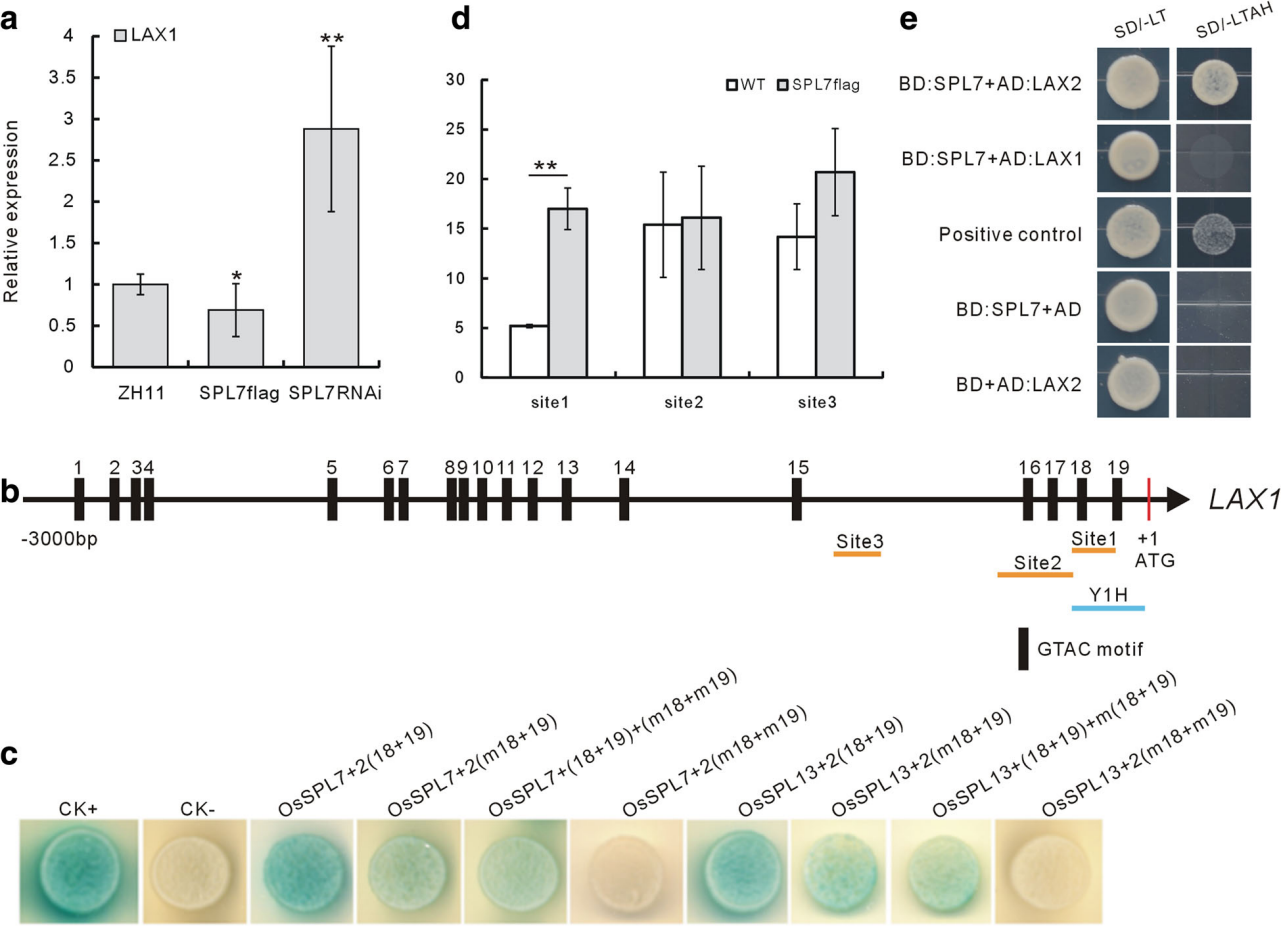
Direct regulation of OsSPL7 to *LAX1* and Y2H of OsSPL7 and LAX2. **a** Expression analysis of *LAX1* gene in the SPL7Flag and SPL7RNAi plants. **b** Sketch graph of the 19 SPL binding motifs in the 2 Kb promoter of *LAX1* gene. Red bar indicates the ATG start codon, and black ones indicate the SPL binding GTAC motifs. Blue line labeled Y1H indicates the fragment site used in Y1H assay. Orange lines indicate the respective sites of the fragments used in ChIP assay. **c** Y1H assay of the binding of OsSPL7 and OsSPL13 proteins to the 18th and 19th motifs in the promoter of *LAX1* gene. m18 and m19 indicate mutant 18th and 19th motifs. 2(18 + 19) means the fragment containing 2 tandem copies of normal 18th and 19th motifs, 2(m18 + 19) means the fragment containing 2 tandem copies of m18 and normal 19th motifs, and (18 + 19) + (m18 + m19) means the fragment containing one copy of normal 18th and 19th motif and one copy of m18 an m19, 2(m18 + m19) means the fragment containing 2 tandem copies of m18 and m19 motifs. **d** ChIP analysis using the binding of the OsSPL7 fused flag tag to the promoter of *LAX1* gene. Single and double asterisks in (**a**) and (**d**) represent significant difference determined by the Student’s *t*-test at *P < 0.05 and **P < 0.01. **e** Y2H assay of OsSPL7 and LAX2, and OsSPL7 and LAX2. “SD-LT” stands for SD media devoid of Leu and Trp amino acids, “SD-LTAH” stands for SD media devoid of Leu, Trp, Ade and His amino acids

miRNAs function through negatively regulating their targets *SPL* genes. In previous study, we had showed that the target gene, *OsSPL7*, mediated miR156f’s regulation on rice plant architecture (Dai et al. 2018). When the fusion protein of OsSPL7 and myc tag was over expressed, the transgenic plants showed phenotype in panicle architecture, with shorter panicle (Fig. 3a, b), more PBs and less SBs (Fig. 3c). Then, we analyzed the expression level of *LAX1* in the *OsSPL7* over expressed SPL7Flag plants, and RNAi plants to see whether *LAX1* expression was also regulated by *OsSPL7* (Dai et al. 2018). Consistent with the results in miR156f transgenic plants (Fig. 1f), *LAX1* gene was down-regulated in the SPL7Flag plants, while up-regulated in the SPL7RNAi plants (Fig. 4a). These data demonstrated that *OsSPL7* gene might also mediated the function of miR156f in regulating *LAX1* expression.

To investigate how OsSPL7 regulated *LAX1* gene expression at the transcriptional level, the promoter sequence of *LAX1* gene was analyzed by searching the database of Plant Cis-acting Regulatory DNA Elements, PLACE, (http://www.dna.affrc.go.jp/PLACE/) to look for the potential SPL binding motifs. In the 3 Kb sequence upstream of the “atg” start codon of *LAX1* gene, there are 19 “GTAC” motifs for potential SPL binding (Fig. 4b). We then checked the binding affinity of the OsSPL7 protein to the 18th and 19th motifs using a yeast one hybrid (Y1H) assay. When two copies of both 18th and 19th motifs were used (“2(18 + 19)” in Fig. 4c), the yeast clones turned sharp blue, when two copies of mutant 18th and normal 19th motifs were used (“2(m18 + 19)” in Fig. 4c), the clones turned faint blue; when one copy of mutant 18th and mutant 19th motif, and one copy of normal 18th and 19th motif were used (“(18 + 19) + (m18 + m19)” in Fig. 4c), the clones were also faint blue, only when two copies of both mutant 18th and mutant 19th motifs were used (“2(m18 + m19)” in Fig. 4c), the clones were not blue any more. Altogether, the Y1H assay demonstrated that the OsSPL7 protein could bind to the 18th and 19th “GTAC” motifs in the *LAX1* promoter region. In addition to OsSPL7, OsSPL13 protein also showed binding ability to the same *LAX1* promoter region in the Y1H assay (Fig. 4c). These direct evidences showed that both miR156f targets, OsSPL7 and OsSPL13, could bind to the promoter region of *LAX1* promoter.

Next, we carried out chromatin immunoprecipitation (ChIP) assay with the transgenic SPL7::SPL7Flag plants, in which OsSPL7 protein was fused with Flag tag and driven by its own promoter (Dai et al. 2018). It was revealed that the DNA fragments of the *LAX1* promoter region were pulled-down by the OsSPL7Flag protein (Fig. 4d). It further confirmed the direct binding of OsSPL7 to the promoter region of *LAX1* gene.

### Genetic analysis revealed interaction between miR156f and *LAX2*

*LAX2* gene encodes a nuclear protein that regulates axillary development, and *lax2* mutant showed similar phenotype as *lax1* mutant (Tabuchi et al. 2011). To investigate the genetic relation between miR156f and *LAX2*, we crossed the MIM156fOE plants to a *lax2* mutant in ZH11 background. The positive F2 hybrid showed less SBs and spikelets (Fig. 2a, b, e, f) compared with the parents, but no change in the number of the PBs as compared with the *lax2* mutant. Then, we analyzed the expression level of *LAX2* gene in the *cs* mutant and the MIM156fOE plants. Consistently, *LAX2* gene was also down-regulated in the MIM156fOE plants and up-regulated in the *cs* mutant (Fig. 1f).

By searching the PLACE database, we also found 10 “GTAC” motifs in the 3 Kb promoter of the *LAX2* gene. However, different from the result from *LAX1*, when we carried out Y1H assay with *LAX2* predicted binding motifs and OsSPLs proteins, there were no binding affinity detected between them (data not shown). It indicated that *LAX2* was not directly regulated by OsSPL7 at the transcriptional level, although they showed genetic interaction. Further, OsSPL7 and LAX2 could directly interact with each other in the yeast two hybrid (Y2H) system but not OsSPL7 and LAX1 (Fig. 4e). In summary, it suggested the direct regulation of OsSPL7 to *LAX1*, but not *LAX2*.

### Genetic analysis revealed interaction between miR156f and *RCN2*

Over expression of several *RCN* genes could greatly increase the number of PBs and SBs (Ikeda-Kawakatsu et al. 2012). According to the microarray data, the expression level of *RCN1*, *RCN3* and *RCN4* were significantly up-regulated in the *cs* mutant (Table 1). We used 2 cm young panicle to perform the qRT-PCR analysis, the expression of all four *RCN* genes were down-regulated in the MIM156fOE plants while up-regulated in the *cs* mutant, and the up-regulation of the *RCN2* and *RCN4* genes was the most dramatic (Fig. 1f). It indicated that miR156f regulated the expression of *RCN* genes, through which the number of panicle branches was influenced.

**Fig. 5:**
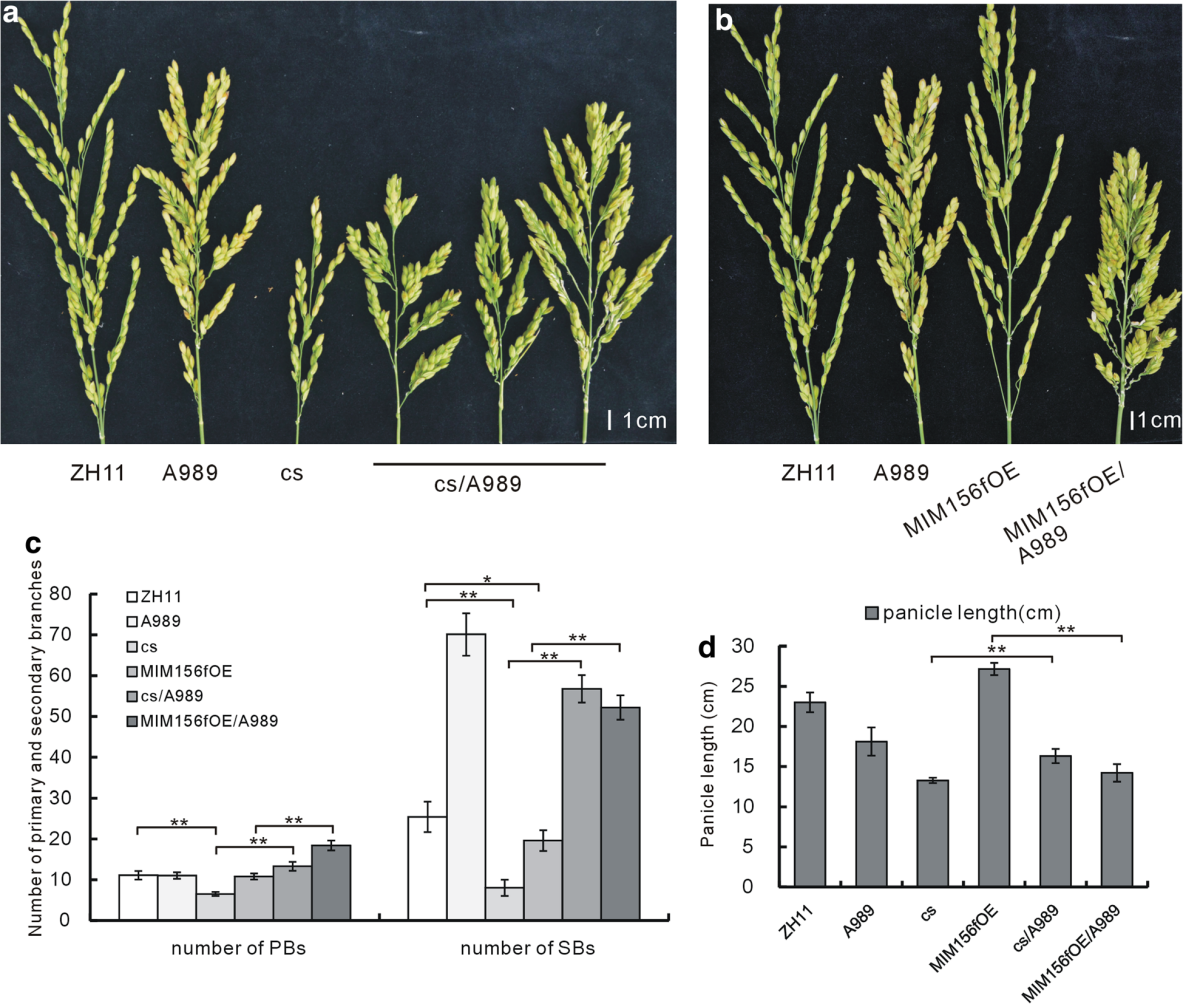
Panicle phenotype of the hybrid of A989 and MIM156fOE, A989 and the *cs* mutant. **a** The panicle morphology of the *cs* and the A989 mutants, their cross, and the WT. **b** The panicle morphology of the MIM156fOE, the A989 mutant, their cross, and the WT. **c** Statistical analysis of the number of PBs and SBs in the A989 mutant, the *cs* mutant, the MIM156fOE plant, the cs/A989 cross, and the MIM156fOE/A989 cross (*n* = 20). **d** Statistical analysis of the panicle length in the A989 mutant, the *cs* mutant, the MIM156fOE plant, the cs/A989 cross, and the MIM156fOE/A989 cross (*n* = 20). Single and double asterisks represent significant difference determined by the Student’s *t*-test at *P < 0.05 and **P < 0.01

In previous study, we had isolated a T-DNA insertion mutant, A989, in which *RCN2* was constitutively over expressed (Li et al. 2010). A989 showed high-density panicle with significantly increased number of PBs and SBs. To investigate the genetic interaction between miR156f and *RCN2* gene, we also crossed A989 with the *cs* mutant and the MIM156fOE plants. The *cs*/A989 hybrid showed longer panicle (Fig. 5a, d) and more PBs and SBs (Fig. 5c) compared with the *cs* mutant. In contrast, the MIM156fOE/A989 hybrid showed shorter panicle (Fig. 5b, d) and more PBs and SBs (Fig. 5c) than the MIM156fOE plants. So that, over expression of *RCN2* increased the number of SBs in both the *cs* mutant and the MIM156OE plants. Other than the panicle number, the plant height and tiller number of the cs/A989 hybrid were similar to the *cs* mutant, while the less tiller number phenotype of the MIM156fOE plant was maintained in the MIM156fOE/A989 plants (Additional file 1: Figure S1).

### Genetic analysis revealed possible interaction between miR156f and *OsRA2*

**Fig. 6:**
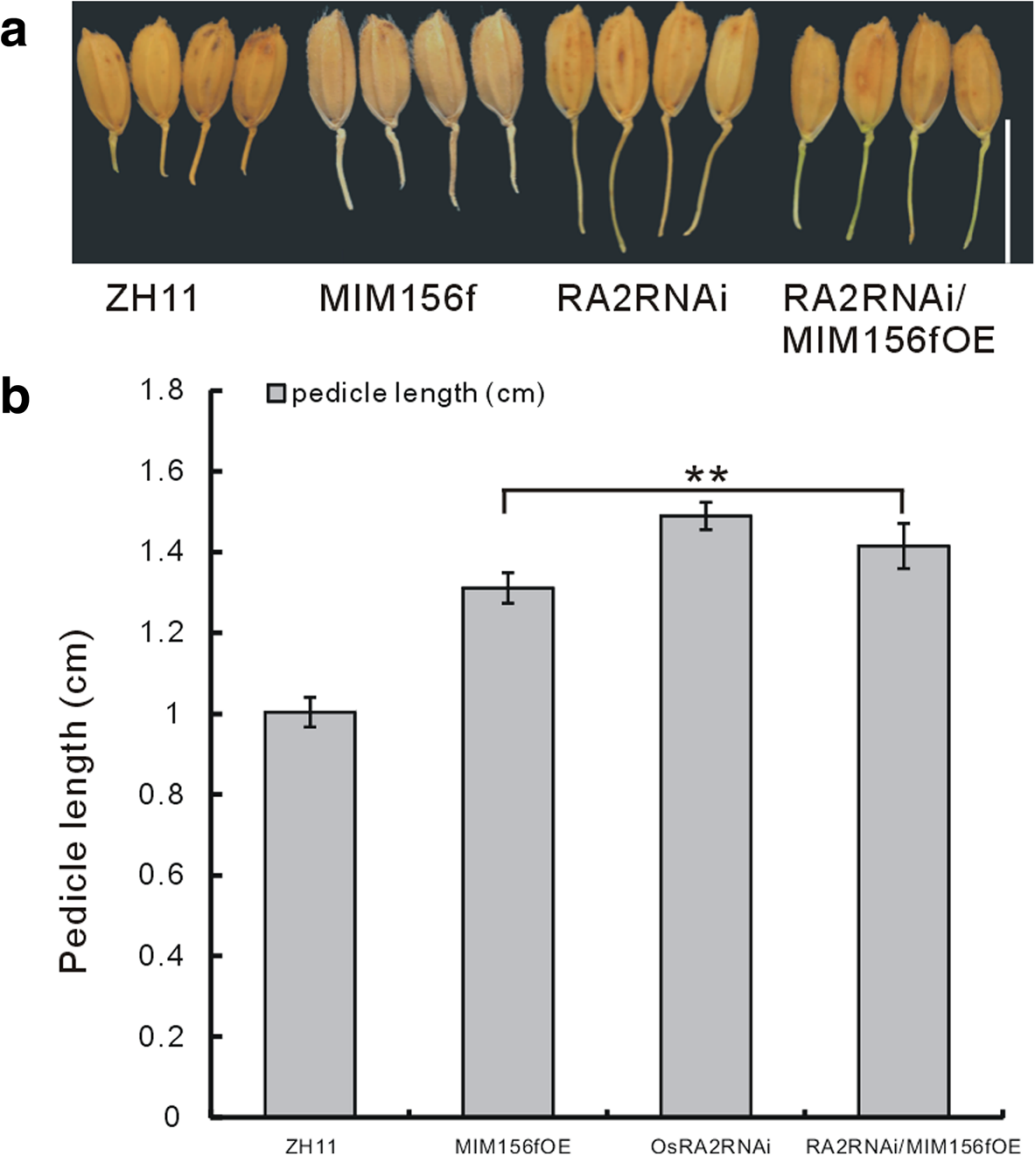
The pedicel phenotype of MIM156fOE crossed with RA2RNAi plants. **a** The morphology of the pedicels of the MIM156fOE, the RA2RNAi plant, their cross and the WT ZH11. bar = 1 cm. **b** Statistical analysis of the pedicel length of the materials in (**a**) (*n* = 20). Single and double asterisks represent significant difference determined by the Student’s *t*-test at *P < 0.05 and **P < 0.01

Since MIM156fOE plants showed elongated pedicels (Fig. 1d), and *OsRA2* gene was reported to have function in the pedicel elongation (Lu et al. 2017), we suscepted that miR156 also had relation with *OsRA2*. To test this hypothesis, we detected the expression level of *OsRA2* in the MIM156fOE plants and the *cs* mutant. Similarly, the expression of *OsRA2* gene was down-regulated in the MIM156fOE plants, while up-regulated in the *cs* mutant (Fig. 1f). The pedicel phenotype of the hybrid from the MIM156fOE and OsRA2RNAi plants was similar to that of the OsRA2RNAi plants (Fig. 6a, b). We also carried out Y1H assay to see whether SPL proteins can directly bind to the promoter regions of *OsRA2* gene, but the result turned out to be negative (data not shown). These results indicated that *OsRA2* might function downstream of miR156f in regulation of pedicel elongation but not through its target *OsSPLs* genes.

## Discussion

Rice panicle is a complex trait under regulation from various aspects including miRNAs (Wang et al. 2015). In this study, we provided genetic evidences to elucidate the relationships between miR156/SPL module and some panicle development related genes, such as *LAX1*, *LAX2*, *RCN2* and *OsRA2*. MIM156OE plants showed decreased number of SBs (Fig. 1c), which were partially due to the down regulation of axillary meristem regulating genes *LAX1* and *LAX2* (Fig. 1f). And these regulations was mediated through the direct binding of OsSPL7 and OsSPL13, the miR156 target genes, to the promoter regions of *LAX1* gene, and interaction between OsSPL7 and LAX2 protein (Figs. 1f and 4). Moreover, *OsSPLs* might have genetic interaction with *LAX2*, and LAX1 and LAX2 could also interact (Tabuchi et al. 2011), thus a triple protein of LAX1\LAX2\SPL complex might be formed (Figs. 2 and 4e). Altogether, axillary meristem regulating genes plays pivotal roles in the miR156f-regulated panicle architecture development. Although *OsSPL14* has been proved to function in ideal plant architecture including panicle (Jiao et al. 2010; Miura et al. 2010), in the *cs* mutant, *OsSPL14* is down-regulated to a degree not that obvious as that of *OsSPL7* or *OsSPL13* (Table 1, Dai et al. 2018), and further analysis proved *OsSPL7* to be the mediating factor of miR156 in regulating *LAX1* expression in this study, this conclusion does not exclude the role of other OsSPLs in panicle trait determination.

miR156f also regulate pedicel length in panicle development (Fig. 1a), with the MIM156fOE plants showing longer pedicel (Fig. 1d). *OsRA2* was reported to regulate pedicel length in rice (Lu et al. 2017). In genetic analysis, it was revealed that miR156f and *OsRA2* functioned in the same pathway in regulating pedicel development (Fig. 6). However, how miR156/OsSPL regulates the expression of *OsRA2* remains unclarified.

In addition, the *RCN2* genes mainly affect the number of PBs and SBs in rice panicle development (Ikeda-Kawakatsu et al. 2012). MIM156fOE plants showed less SBs (Fig. 5b, c), this phenotype was restored in the MIM156fOE/A989 hybrid by over expression of *RCN2* (Fig. 5b, c), indicating that miR156 regulated the number of branches in the panicle through *RCN2*. In the *cs* mutant, where *RCN2* was up-regulated (Fig. 1f), the panicle was not dense, however, when crossed with A989, the panicle was dense (Fig. 5a, c). Indicating that regulation of *RCN2* on the number of branches might have dosage effect. An example for this is that when *RCN2* was driven by double 35S promoter, the panicle failed to develop, which might due to too much delayed spikelet meristem transition (Li et al. 2010).

Theoretically, the *cs* mutant would show a much dense panicle, with increased expression of several *RCN* genes which would increase the number of PBs and SBs; increased expression of *OsRA2*, which would decrease pedicel length, and increased expression of *IDS1* and *MFS1*, which would increase the number of spikelets (Fig. 1f). However, the *cs* mutant showed a panicle architecture quite to the contrary (Fig. 1c, e). One explanation is that there might be some buffering mechanism under control of miR156f, since if the *cs* mutant develop accordingly to the expression level of these panicle related genes, it would be an extreme dense panicle that run out of control, not to mention that many genes have dosage effect. This buffering mechanism might be a result of the interwoven interaction among different panicle development related factors, which deserve more investigation.

## Conclusions

We revealed that miR156f influenced expression of many panicle related genes. miR156f/SPL directly regulated *LAX1* at the transcription level. And tight genetic relations existed between miR156f and *LAX1*, and miR156f and *LAX2*. Meanwhile, there was genetic relation between miR156f and *RCN2*, which might provide a buffering mechanism for miR156f in mediating panicle architecture regulation. miR156f might function in the same pathway with *OsRA2* in pedicel regulation. We concluded that miR156f acts as an important regulator of panicle architecture through influencing various aspects of panicle development.

## Material and methods

### Plant species and growth conditions

Wild type rice species ZH11 (*Orayza sativ*a *L. subsp*. japonica cv. Zhonghua No. 11) was used as the host for transgenic transformation in this study. ZH11, the *cs* mutant the A989 mutant, the *lax1* mutant, the *lax2* mutant, and all the transgenic plants were grown in the green house, with 10 h light and 14 h dark, or in the field under natural conditions in summer, Shanghai, China. The construction of the respective transgenic plants was described in respective references.

### qRT-PCR analysis

Total RNA was extract from different tissues using TRIzol (Invitrogen), followed by DNase I digestion. For qRT-PCR, cDNA was synthesized from 1 μg of total RNA using One Step SYBR PrimeScript RT-PCR Kit (TaKaRa), and 1 μl of cDNA was used as template for real-time analysis. Sampling and expression measurement was repeated three times. The *actin* gene was used as internal reference.

### Y1H assay

The full-length cDNAs of *OsSPL7* and *OsSPL13* were amplified with gene-specific primers (Additional file 2: Table S1), and then fused into the activation-domain (AD) of vector pPC86. Fragments containing “GTAC” in *lax1* gene promoter were amplified with gene-specific primers (Additional file 2: Table S1) and fused into the vector p178 at the *Xho*I site. The p178 and pPC86 constructs were transformed into the yeast strain EGY48 together. The yeast strain was growth on SD selective medium (SD-His-Leu) and observed in blue on Chromogenic medium. The transformants containing void plasmid pPC86 and p178 constructs were used as a negative control. Y1H assay was carried out as described (Matchmaker One-hybrid System; Clontech).

### Y2H assay

The open reading frame (ORF) of *OsSPL7* was amplified and cloned into the prey vector pGAD-T7. The ORFs of *LAX1* and *LAX2* were amplified and cloned into the bait vector pGBK-T7. The Y2H assay was performed according to the manufacturer’s instructions (Clontech).

### ChIP analysis

ChIP analysis was carried out as previoulsy described (Dai et al. 2018).

### Measurement of panicle traits

At least 20 panicles from each line were used for the analysis of panicle characters, including panicle length, the number of PBs and SBs. Fifty spikelets from each line were used to analyze the pedicel length. Data was shown as mean ± SD.

### Measurement of plant height and tiller number

Plant height of at least 20 plants were measured at the mature stage, and effective tillers (tillers that bear panicles) were counted at the same time. Data was shown as mean ± SD.

## Additional files

Additional file 1: Plant height and tiller number of the cross between A989 and MIM156fOE plants, and A989 and the cs mutant. (TIF 12961 kb)

Additional file 2: Primer sequences used in this study. (XLSX 11 kb)

## Data Availability

The datasets used and/or analyzed during the current study are available from the corresponding author on reasonable request.

## Abbreviations

AM: Axillary meristem
CK: Cytokinin
DST: DROUGHT AND SALT TOLERANCE
*FZP*: *FRIZZY PANICLE*
GA: Gibberellin
IM: Inflorescence meristem
*LAX1*: *LAX PANICLE 1*
*LAX2*: *LAX PANICLE 2*
*LP*: *LARGER PANICLE*
MIM156f: Mimicry miR156f
MIM156fOE: Mimicry miR156f over-expression
miRNA: microRNA
*MOC1*: *MONOCULM1*
*OsCKX2*: *CYTOKININ OXIDASE*
*OsRA2*: *OsRAMOSA2*
PB: Primary branches
SAM: Shoot apical meristem
SAM: Shoot apical meristem
SB: Secondary branches
SM: Spikelet meristem
*SPL*: *SQUAMOSA PROMOTER BINDING PROTEIN-like*
SPL7Flag: *OsSPL7* fused with flag tag driven by its own promoter
SPL7myc: *OsSPL7* fused with myc tag driven by its own promoter
SPL7RNAi: *OsSPL7* RNA interference
