# Supplementary figures and images for: miR156f integrates panicle architecture through genetic modulation of branch number and pedicel length pathways
Source: Rice (N Y). 2019 May 30;12:40. doi: 10.1186/s12284-019-0299-5 (PMC6542935; doi:10.1186/s12284-019-0299-5)

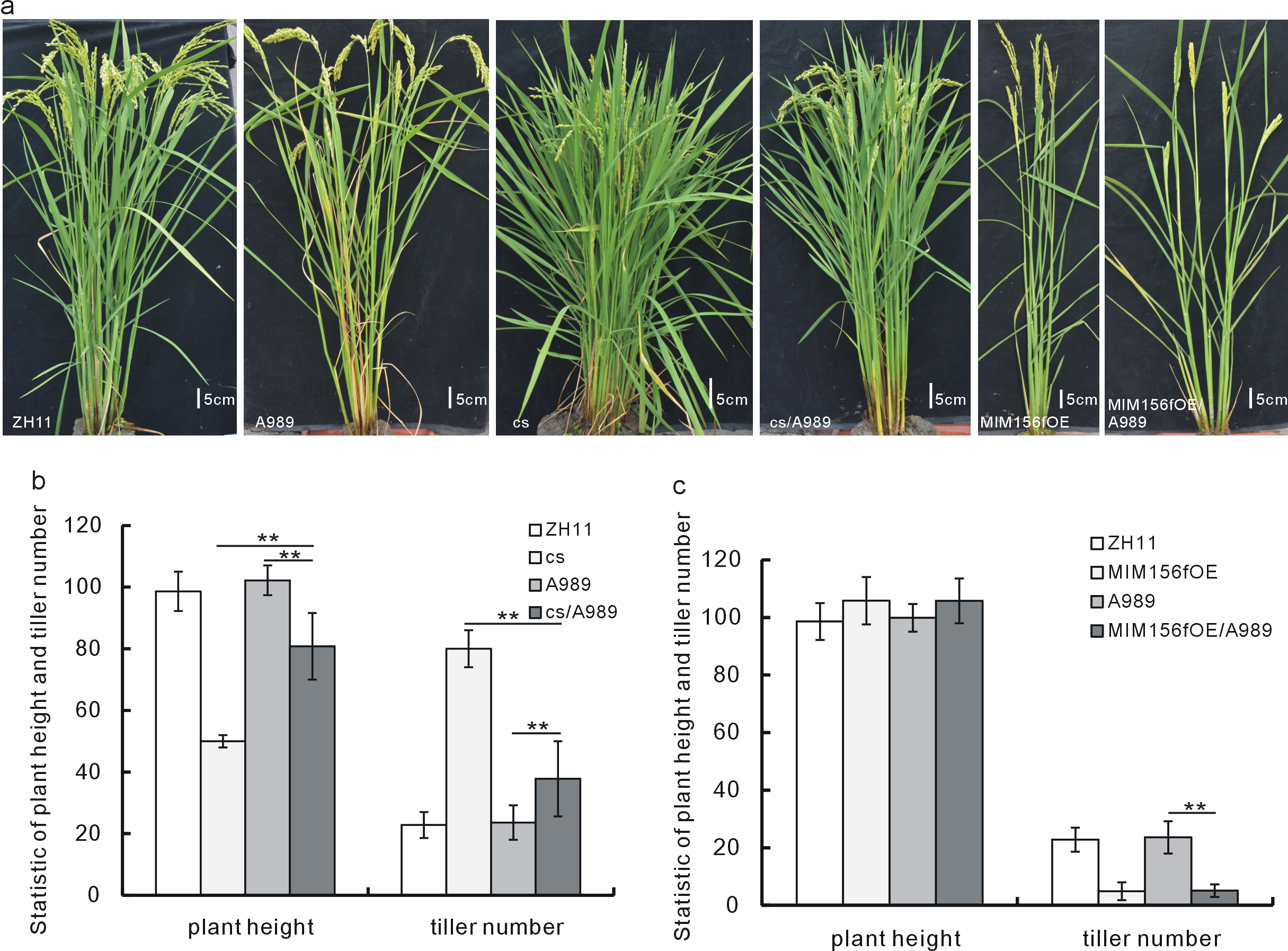

Supplement: Supplementary file 1 — Plant height and tiller number of the cross between A989 and MIM156fOE plants, and A989 and the cs mutant. (TIF 12961 kb) [file 12284_2019_299_MOESM1_ESM.tif]
